# Varenicline induced auditory hallucinations in a young female with bipolar disorder: a case report

**DOI:** 10.1192/j.eurpsy.2023.1610

**Published:** 2023-07-19

**Authors:** S. Bhanot, V. W. L. Tsang, L. Jia

**Affiliations:** 1 Biochemistry and Biomedical Sciences, McMaster University, Hamilton; 2 Department of Psychiatry, University of British Columbia, Vancouver, Canada

## Abstract

**Introduction:**

Creating appropriate and sustainable treatment plans for patients with concurrent disorders presents a challenge to psychiatrists and addiction medicine specialists alike. Although varenicline has been found to be one of the most effective medications for smoking cessation and abstinence, caution is needed when starting patients on this medication. In this case, a young female provisionally diagnosed with bipolar I disorder was hospitalized for a manic episode in the context of substance abuse and medical noncompliance. She also endorsed a long history of smoking, alcohol, cocaine, cannabis and ketamine use. In addition to being stabilized for bipolar disorder, the patient was started on varenicline for smoking cessation on Day 14 of admission.

**Objectives:**

This case report highlights the potential risk of de-stabilization in a vulnerable youth with newly diagnosed bipolar I disorder and precarious social circumstances, in attempts to further concurrent approaches to psychiatric care.

**Methods:**

In addition to qualitative observations, the main objective exam used to track patient progress through the duration of her hospitalization was the mental status exam (MSE). This is standard practice for psychiatric care and qualitatively assessed factors related to a patient’s behavioral and cognitive functioning. Important factors assessed for this patient include appearance and behavior, speech, affect and mood, thought form, thought content, perceptual abnormalities, insight and cognition.

**Results:**

Perceptual abnormalities, including auditory hallucinations, were not recorded at admission and the patient’s symptoms of mania were resolving clinically on Day 18. Two days after starting varenicline, the patient developed auditory hallucinations, paranoia and referential beliefs. However, her insight was intact, and she had minimal thought-form disorganization. The patient also reported hearing auditory hallucinations of a derogatory nature, with her mood appearing more distressed during varenicline use. Symptoms were found to be resolved shortly after the discontinuation of varenicline on Day 18 and the patient appeared to be less distressed on following days. In this case, these symptoms were not in keeping with her bipolar diagnosis and thought to be secondary to varenicline after the consideration of potential alternative contributors.

**Conclusions:**

The occurrence of side effects as a result of varenicline use in patients with diagnosed mental health conditions is rare and underlying psychiatric illness is not labeled as an absolute contraindication in the prescription of varenicline. However, it is important to advocate for increased guidance and research on the treatment of substance use disorders in patients with bipolar I disorder.

**Disclosure of Interest:**

None Declared

